# A Comprehensive Investigation to Reveal the Relationship Between Plasmacytoid Dendritic Cells and Breast Cancer by Multiomics Data Analysis

**DOI:** 10.3389/fcell.2021.640476

**Published:** 2021-04-01

**Authors:** Saisai Tian, Li Yan, Lu Fu, Zhen Zhang, Jinbo Zhang, Guofeng Meng, Weidong Zhang

**Affiliations:** ^1^School of Pharmacy, Second Military Medical University, Shanghai, China; ^2^Institute of Interdisciplinary Integrative Medicine Research, Shanghai University of Traditional Chinese Medicine, Shanghai, China; ^3^Department of Pharmacy, Tianjin Rehabilitation and Recuperation Center, Joint Logistics Support Force, Tianjin, China

**Keywords:** breast cancer, immunotherapy, plasmacytoid dendritic cells, prognosis, bioinformatics analysis

## Abstract

Plasmacytoid dendritic cells (pDC) are an essential immune microenvironment component. They have been reported for crucial roles in linking the adaptive and immune systems. However, the prognostic role of the pDC in breast cancer (BRCA) was controversial. In this work, we collected large sample cohorts and did a comprehensive investigation to reveal the relationship between pDC and BRCA by multiomics data analysis. Elevated pDC levels were correlated with prolonged survival outcomes in BRCA patients. The distinct mutation landscape and lower burden of somatic copy number alterations (SCNA) and lower intratumoral heterogeneity were observed in the high pDC abundance group. Additionally, a more sensitive immune response and chemotherapies response were observed in the high pDC group, which implicates that patients with high pDC abundance can be benefited from the combination of chemotherapy and immunotherapy. In conclusion, the correlation between pDC abundance and BRCA patients’ overall survival (OS) was found to be positive. We identified the molecular profiles of BRCA patients with pDC abundance. Our findings may be beneficial in aiding in the development of immunotherapy and elucidating on the precision treatment for BRCA.

## Introduction

Breast cancer (BRCA) is a pathological state in which breast epithelial cells proliferate out of control under the action of multiple carcinogens. The International Agency for Research on Cancer (IARC) documented that in 2018, BRCA has an incidence rate of 24.2% among females globally and is the most susceptible malignant tumor for women. There are four breast subtypes: lumina A (cavity surface A), lumina B (cavity surface B), HER-2 positive, and triple-negative. Although the overall survival (OS) for early BRCA patients continues to increase, the vast majority of advanced BRCA patients cannot be cured. As a new star in the field of oncology, immunotherapy has drawn lots of attention. In the treatment of solid tumors, such as liver cancer, lung cancer, pancreatic cancer, and ovarian cancer, immunotherapy has been shown to exert a strong anti-tumor activity (Steven et al., 2016; Odunsi, 2017; Banerjee et al., 2018; Emens, 2018). The Food and Drug Administration (FDA) has approved several tumor immunotherapy drugs for clinical application (Riley et al., 2019). Moreover, immunotherapy has been used for advanced BRCA, but only for specific subtypes, such as programmed death-ligand 1 (PD-L1) monoclonal antibody atezolizumab for triple-negative BRCA. Phase III clinical studies have confirmed that the combination of chemotherapy and immunotherapy can improve the curative effect and improve the OS (Schmid et al., 2018).

However, only relying on a PD-L1 test will definitely miss some patients who can also benefit from immunotherapy. Concurrently, current research enthusiasm concentrated on T cells, and less attention paid to other types of immune cells. Hollern’s team confirmed for the first time that the production of antibodies by B cells plays a crucial role in anti-tumor immune response (Hollern et al., 2019). This shows that different immune cell types have unique importance in the immune system and immunotherapy still needs to seek more breakthroughs for general patients with BRCA. Based on lineage-negative cells with dendritic cell (DC)-like morphology (Chehimi et al., 1989; O’Doherty et al., 1993), upon stimulation with influenza or herpes simplex viruses, the plasmacytoid dendritic cells (pDC) were found to be the key type I interferon (IFN)-secreting cells in circulation (Cella et al., 1999; Siegal et al., 1999). Therefore, these cells play a crucial role in adaptive and innate immune defenses against viruses, other pathogens, and autoimmune diseases as well as in anti-tumor immunity.

However, the role of the pDC is different among different tumor types (Han et al., 2017; Schuster et al., 2019; Wagner et al., 2019; Zhou et al., 2019). Studies on the role of the pDC on the clinical outcomes of BRCA patients have not found a binding conclusion (Sawant et al., 2012; Sisirak et al., 2012, 2013; Faget et al., 2013; Sawant and Ponnazhagan, 2013; Kini Bailur et al., 2016; Wu et al., 2017; Gadalla et al., 2019). This study aimed at evaluating the role of the pDC in BRCA patients. First, we measured the tumor-infiltrating immune cells (TIICs) of BRCA by the Single-Sample Gene Set Enrichment Analysis (ssGSEA) algorithm and constructed the interaction networks of the TIICs. Then, we conducted a detailed analysis to determine the potential role of the pDC from a multiomics view including somatic copy number alterations (SCNA), burden of SCNA, somatic mutation, and intratumoral heterogeneity. Finally, we revealed the influences of the pDC in BRCA and offered potential therapeutic strategies for the precise treatment of different molecular subtypes of BRCA. In short, we evaluated the roles of the pDC, pDC-associated genes, as well as immunotherapeutic outcomes in BRCA using machine learning and bioinformatics models. Our findings reveal probable therapeutic targets and elucidate on the molecular mechanisms of the BRCA microenvironment.

## Materials and Methods

### Patients and Samples

**FIGURE 1:**
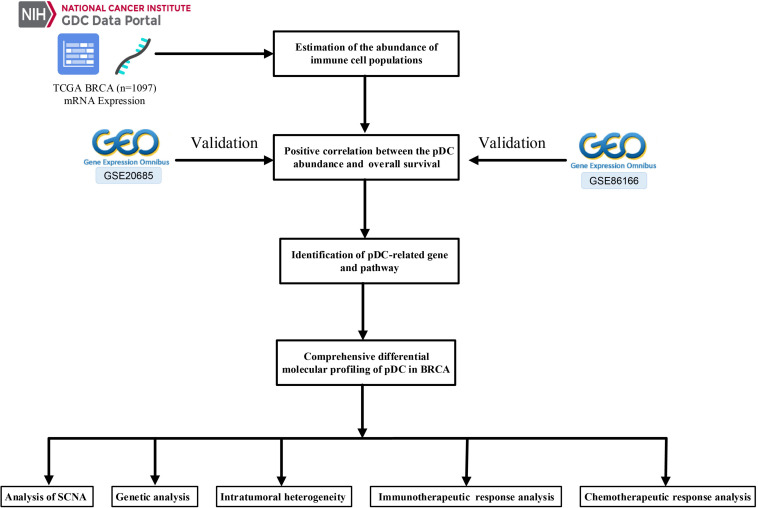
Schematic presentation of the study design.

The Cancer Genome Atlas (TCGA)-BRCA mRNA count data and their relevant clinical information were retrieved from the GDC data portal^1^. In this paper, 1,097 BRCA samples with corresponding clinical information were available in TCGA. Simultaneously, the somatic mutation data (VarScan2 Variant Aggregation and Masking) were retrieved from TCGA database. Moreover, SCNA were downloaded from the Firehose database^2^. Another two independent cohorts, including GSE20685 and GSE86166, were obtained from the Gene Expression Omnibus (GEO) database^3^ to confirm the performance of the prognostic pDC. The detailed TCGA clinical information is shown in Table 1. Figure 1 shows the workflow of this study, that is, data pre-processing, estimation of the immune cell abundance, analysis of SCNA, and the immunotherapeutic and chemotherapeutic response prediction.

**TABLE 1:** Clinical characteristics of BRCA patients in TCGA.

Characteristic	TCGA
Age	
Median	58
Range	19–90
Sex	
Male	12
Female	1,085
M stage	
M0	907
M1	190
N stage	
N0	514
N1	365
N2	120
N3	78
NX	20
T stage	
T1	280
T2	637
T3	137
T4	40
Unknown	3
pTNM stage	
Stage I	181
Stage II	625
Stage III	250
Stage IV	20
Stage X	13
Unknown	8
Vital status	
Living	943
Dead	154

### Pre-Processing of Gene Expression Data

We first transformed the Ensembl IDs to gene symbols and preserved protein-coding genes. Given that transcripts per kilobase million (TPM) values were similar to the results generating from microarray, we computed the TPM values. For the GEO datasets, we normalized the expression datasets by Robust Multiarray Average with the R package “affy” (Gautier et al., 2004). Mean value was selected in cases where multiple probes were mapped to the same gene.

### Estimation of the Abundance of Immune Cell Populations

Gene signatures of 24 TIICs, including adaptive and innate immunity, were used to calculate the infiltration level of immune cell through the ssGSEA algorithm (Bindea et al., 2013). In brief, ssGSEA applied the special signatures of immune cells to determine the abundance of immune cell populations in every sample. In our research, we did ssGSEA to estimate the infiltration levels of 24 kinds of TIICs in BRCA samples, which was implemented in the R package GSVA (Hänzelmann et al., 2013). The correlations and heatmap between the infiltration levels of the TIICs were established to show the relationship between the TIICs using the corrplot and ComplexHeatmap packages (Gu, 2015; Wei et al., 2017).

### Survival Analysis of TIICs Abundance of BRCA

After establishing the abundance of the TIICs in BRCA samples by the GSVA package, we further identified whether the TIICs have prognostic value. For TCGA and GEO datasets, the TIICs’ infiltration levels data of patients and clinical data are combined into a matrix. Then, using the survminer package in R (Kassambara et al., 2017), we determined the optimal cut-off point for group division. Using the cut-off value, the samples were assigned into the pDC^high^ group and the pDC^low^ group. Finally, using the survival package (Therneau and Lumley, 2014; Tian et al., 2019b), the R software was used to show the influence of the abundance of the TIICs on OS. Given that the pDC play crucial roles in investigating adaptive and innate immune responses, the results also showed that the tumor-infiltrating pDC were positively correlated with OS; therefore, we aimed at evaluating the effect of the pDC on the BRCA microenvironment.

### Identification of Differentially Expressed Genes Associated With the pDC

In TCGA dataset, we used the limma package to calculate the differentially expressed genes (DEGs) between the pDC^high^ and pDC^low^ groups at the cut-off | logFC| > 0.585 and adj.P.Val < 0.05 (*p*-value was attuned for multiple tests using the Benjamini–Hochberg method) (Smyth, 2005; Tian et al., 2019b). The heatmap and volcano map were drawn, where highly expressed genes were marked in red, whereas genes with suppressed expression levels were marked in blue. The Gene Ontology (GO) and Kyoto Encyclopedia of Genes and Genomes (KEGG) analyses were performed for gene set annotation, whereas the GSEA algorithm was applied to identify the key pathways and biological process of the pDC-related genes using the R package “clusterProfiler” (Yu et al., 2012; Tian et al., 2019a).

### Survival Analysis of Hub Genes Based on the DEGs

For the DEGs between the pDC^high^ and pDC^low^ groups, we used the Search Tool for the Retrieval of Interacting Genes (STRING, version 11.0)^4^ online database with the cut-off criterion of combined score > 0.4 to establish the protein–protein interaction (PPI) networks (Mering et al., 2003). In addition, three network topology parameters, including degree, betweenness, and closeness, were used to filter the key genes, and the top 10 key gene relationship networks were built using cytoHubba plugin (Chin et al., 2014). Finally, a Venny map was built to show the hub gene, and survival analysis also was performed to show whether the hub genes have prognostic value.

### Analysis of SCNA

SCNA data of BRCA were downloaded from Firehose^5^ and, based on pDC abundance, were distributed into two groups. The GISTIC 2.0 module of GenePattern (Mermel et al., 2011) was used to analyze the SCNA data of the pDC^high^ group and the pDC^low^ group. In this paper, the hg19.mat data with a threshold of 0.1 were selected. In addition, we calculated the burden of SCNA including gain as well as loss at the focal and arm levels.

### Genetic Analysis and Intratumoral Heterogeneity

The MutSigCV_v1.41^6^ was used in the identification of significantly mutated genes (*q* < 0.05) more than expected by chance with default parameters (Lawrence et al., 2013). Fisher’s exact test was used to discover differentially mutated genes between the pDC^high^ and pDC^low^ groups. The R package “ComplexHeatmap” was used to draw the mutation landscape oncoprint. Concurrently, we also calculated the intratumoral heterogeneity score by the R package maftools (Mroz and Rocco, 2013; Mayakonda et al., 2018). The specific calculation formula was shown as:

MATH=100×MAD/median.

The MATH score of each individual was calculated based on median of mutant-allele fractions and median absolute deviation (MAD). In somatic mutations, the MATH score could quantify the genetic heterogeneity by the normalized variance of the frequency distribution of mutant alleles.

### Immunotherapeutic and Chemotherapeutic Response Prediction

Using two prediction methods, we further explored the possibility of clinical responses to immune checkpoint blockade. The Tumor Immune Dysfunction and Exclusion (TIDE) algorithm as well as the subtype mapping method were employed to evaluate each sample likelihood of responding to immunotherapy (Hoshida et al., 2007; Jiang et al., 2018). Moreover, using the R package “pRRophetic,” chemotherapeutic responses of each sample were predicted using the largest public pharmacogenomics database, that is, the Genomics of Drug Sensitivity in Cancer (GDSC) database^7^ (Geeleher et al., 2014b). Based on the GDSC dataset, the pRRopheticPredict function was used to predict the half-maximal inhibitory concentration (IC50) of each sample using ridge regression. The 10-fold cross-validation method was used to evaluate the prediction accuracy. Using ComBat function, we eliminated batch effects between cell lines for analysis (Geeleher et al., 2014a).

### Statistical Analysis

Statistical analysis was performed using the R software. The survival package was used for survival analysis. The difference of OS was assessed using Kaplan–Meier plots and log-rank tests. The correlation between defined groups and categorical clinical information was assessed using chi-square. *p* ≤ 0.05 was set as the threshold for statistical significance.

## Results

### High pDC Abundance in BRCA Is Correlated With Better Survival Outcomes

**FIGURE 2:**
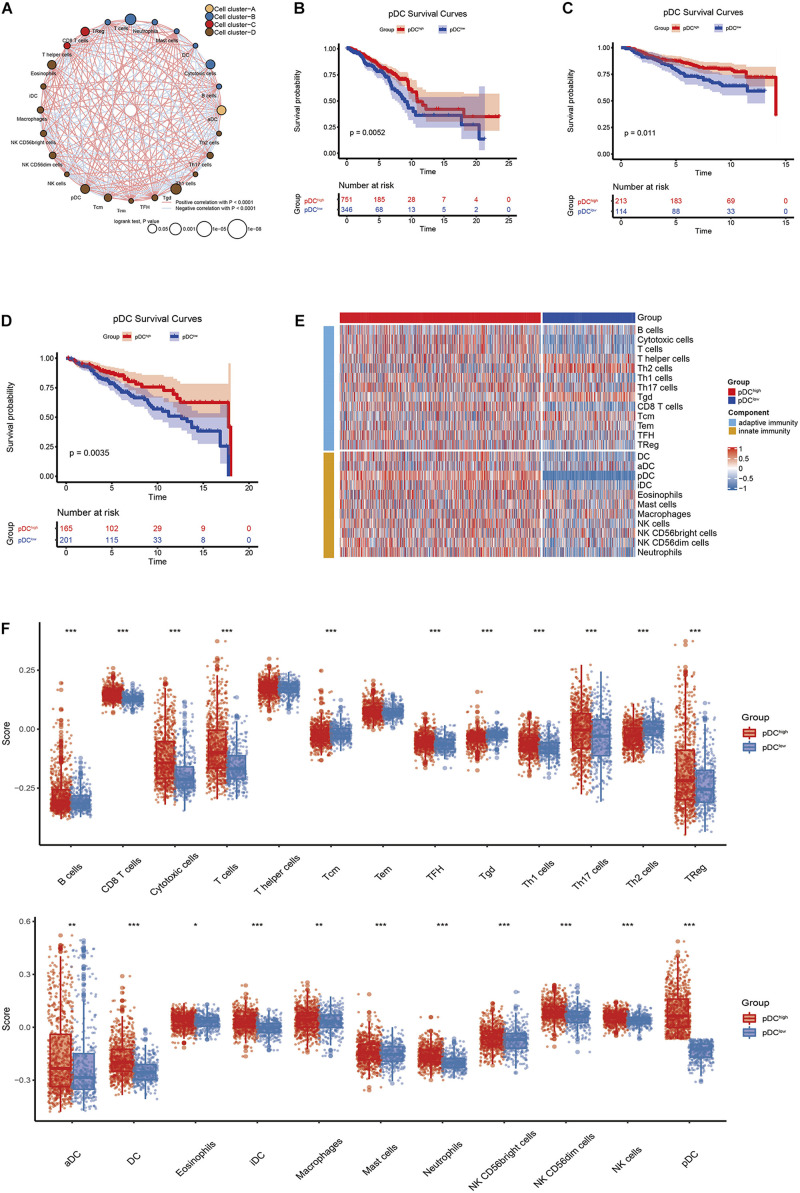
The correlation between clinical outcomes and pDC abundance in BRCA patients. **(A)** Correlations among immune cell populations. **(B–D)** Kaplan–Meier curves for the OS of BRCA patients revealed that patients with elevated pDC abundance exhibited favorable clinical outcomes when compared with patients with low pDC abundance in TCGA, GSE20685, and GSE86166 cohorts. **(E,F)** The immune infiltration levels between the two groups, including adaptive immune signatures and innate immune signatures. The meaning of the statistical difference is as follows: * represents *p* ≤ 0.05, ** represents *p* ≤ 0.01, and *** represents *p* ≤ 0.001.

Figure 1 shows the workflow of the whole analysis. First, the ssGSEA approach was used to calculate the richness of 24 immune cell populations in BRCA samples. In order to show the immune phenotype landscape in the tumor microenvironment (TME) of BRCA, the TME cell correlations network and their effects on the OS of patients were constructed by hierarchical cluster analysis. As is shown in Figure 2A, the TME immune cells, drawing an overall landscape of TME interactions, were clustered into four clusters, and the relationship between OS and clusters was analyzed by the pairwise log-rank test. Using an optimal cut-off value determined by the survminer package in R, we divided BRCA samples into the pDC^high^ group and the pDC^low^ group. Specifically, the pDC^high^ group, comprising 751 samples, was distinguished from the pDC^low^ group, containing 346 samples. The survival analysis suggested that the abundance of the pDC was strongly positively associated with patient’s clinical outcome, which means that the elevated abundance of the pDC benefited the survival outcomes of BRCA patients included in TCGA cohorts (Figure 2B). Additionally, two external cohorts (GSE20685 and GSE86166) were used to verify the association between the pDC and the survival of BRCA patients (Figures 2C,D). In addition, adaptive immunity and innate immunity were compared. The pDC^high^ group was highly enriched in adaptive and innate immunity (Figures 2E,F).

### Identification and Functional Enrichment Analysis of the DEGs Associated With the pDC

**FIGURE 3:**
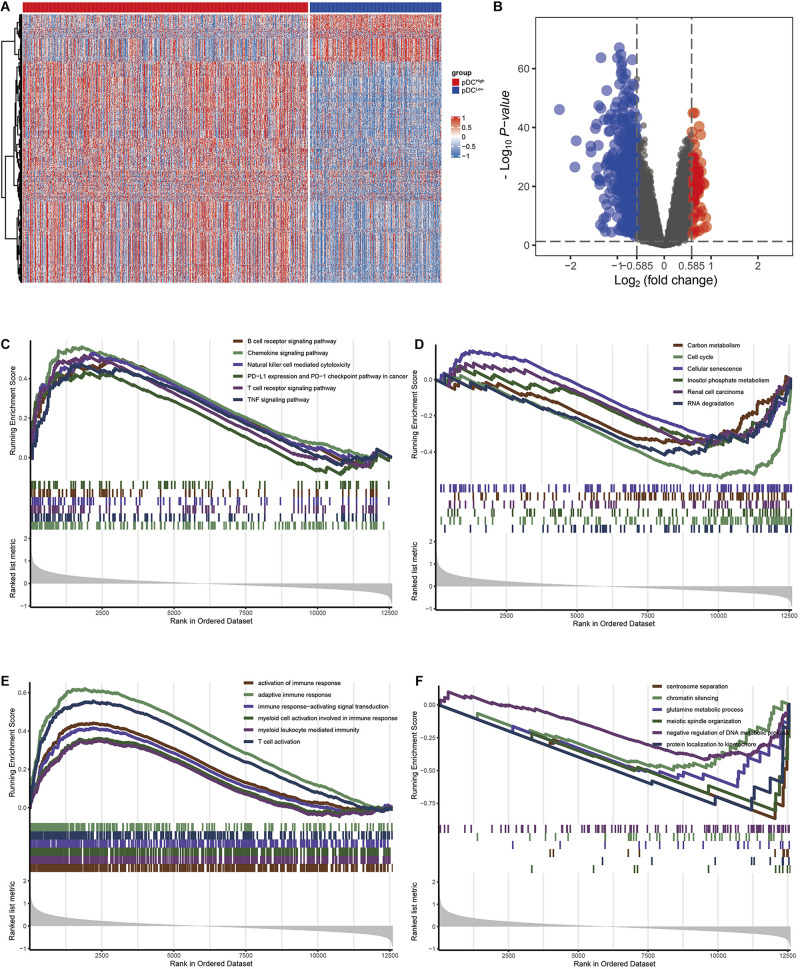
The DEGs and pathways between the two groups. **(A)** Heatmap of the DEGs between the two groups. **(B)** The volcano plot of the DEGs between the two groups. **(C)** KEGG analysis in the pDC^high^ group. **(D)** KEGG analysis in the pDC^*low*^ group. **(E)** GO analysis demonstrated in the pDC^high^ group. **(F)** GO analysis in the pDC^*low*^ group.

Using the “limma” R package, we performed a differential expression analysis of two pDC groups at the cut-off |logFC| > 0.585 and adj.*P*.Val < 0.05 in TCGA. Then, 542 DEGs (95 down-regulated and 447 up-regulated genes) were identified for further analysis in TCGA. The heatmap and volcano plots of the DEGs are shown in Figures 3A,B. In order to characterize the pathway differences between the two groups, KEGG and GO were utilized for functional enrichment analysis by GSEA. The results showed that the pDC^high^ group was enriched with immune-related pathways, such as T cells, B cells, natural killer (NK) cells, PD-L1, chemokines, and tumor necrosis factor (TNF) signaling pathways, and biological processes, such as activation of immune response, T cell activation, and adaptive immune responses, whereas the pDC^*low*^ group was enriched with cellular senescence, cell cycle, renal cell carcinoma, and several metabolic processes (e.g., carbon metabolism, inositol phosphate metabolism) (Figures 3C–F). The detailed parameters of the related results are shown in Tables 2, 3, which indicate that these two groups play different roles—therefore, they have different effects on the prognosis of patients.

**TABLE 2:** The enriched KEGG pathways associated with the pDC in TCGA.

ID	Description	Set size	NES	*p* value	Group
hsa04662	B cell receptor signaling pathway	74	1.961	0.001	pDC^high^
hsa04062	Chemokine signaling pathway	158	2.404	0.001	pDC^high^
hsa04650	Natural killer cell-mediated cytotoxicity	86	2.08	0.001	pDC^high^
hsa05235	PD-L1 expression and PD-1 checkpoint pathway in cancer	76	1.688	0.001	pDC^high^
hsa04660	T cell receptor signaling pathway	90	2.048	0.001	pDC^high^
hsa04668	TNF signaling pathway	101	1.893	0.001	pDC^high^
hsa01200	Carbon metabolism	92	−1.659	0.003	pDC^low^
hsa04110	Cell cycle	121	−2.587	0.004	pDC^low^
hsa04218	Cellular senescence	143	−1.639	0.004	pDC^low^
hsa00562	Inositol phosphate metabolism	70	−1.637	0.007	pDC^low^
hsa05211	Renal cell carcinoma	62	−1.531	0.016	pDC^low^
hsa03018	RNA degradation	67	−1.818	0.003	pDC^low^

**TABLE 3:** The enriched GO pathways associated with the pDC in TCGA.

ID	Description	Set size	NES	*p* value	Group
GO:0002253	Activation of immune response	451	2.078	0.001	pDC^high^
GO:0002250	Adaptive immune response	294	2.806	0.001	pDC^high^
GO:0002757	Immune response-activating signal transduction	400	1.937	0.001	pDC^high^
GO:0002275	Myeloid cell activation involved in immune response	445	1.698	0.001	pDC^high^
GO:0002444	Myeloid leukocyte-mediated immunity	450	1.643	0.001	pDC^high^
GO:0042110	T cell activation	374	2.579	0.001	pDC^high^
GO:0051299	Centrosome separation	10	–2.225	0.002	pDC^low^
GO:0006342	Chromatin silencing	40	–1.924	0.003	pDC^low^
GO:0006541	Glutamine metabolic process	18	–1.930	0.003	pDC^low^
GO:0000212	Meiotic spindle organization	10	–2.072	0.002	pDC^low^
GO:0051053	Negative regulation of DNA metabolic process	101	–1.942	0.004	pDC^low^
GO:0034501	Protein localization to kinetochore	10	–2.059	0.002	pDC^low^

### Screening for Hub Genes

**FIGURE 4:**
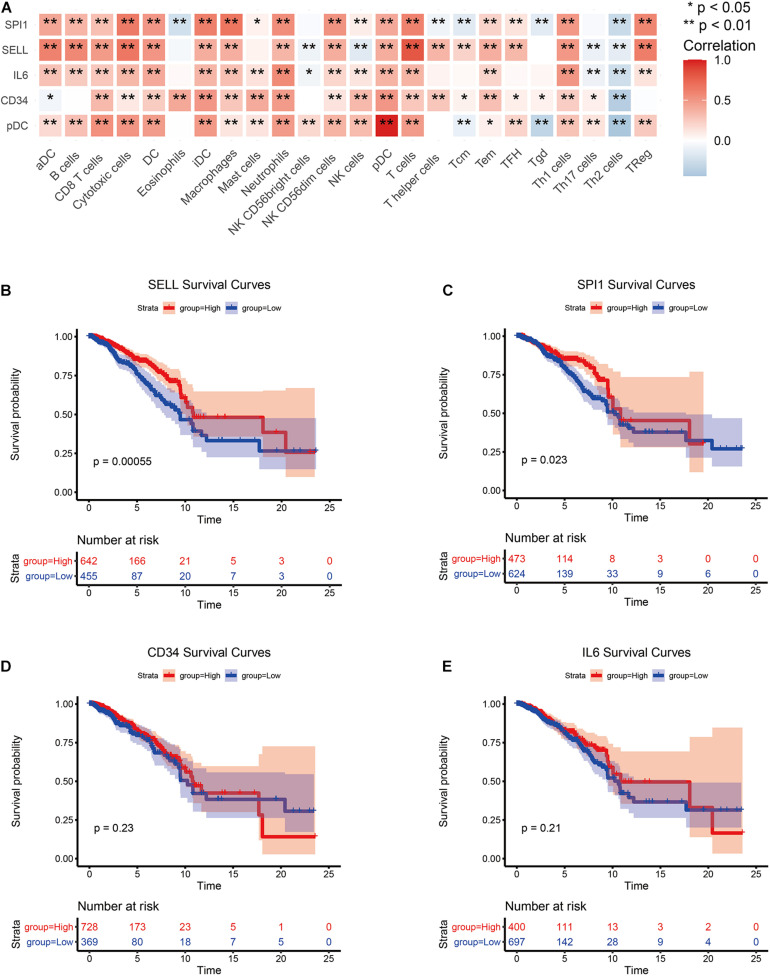
Screening of hub genes and correlation analysis. **(A)** Correlation analysis between hub genes and TIICs. **(B–E)** Survival analysis of hub genes.

The PPI network of all DEGs was established based on the network annotation of the STRING database under default parameters. The network, which consisted of 499 nodes and 4,353 edges, was visualized using the cytoscape software^8^ (Supplementary Figure 1). Then, using the top 10 key genes, three subnetworks were built by three network topology parameters, including degree, betweenness, and closeness. The cytoHubba plugin was used to select the three subnetworks of PPI, and the results are shown in Supplementary Figure 2. Four hub genes, including CD34, IL6, SPI1, and SELL, were selected (Supplementary Figure 3). Then, we examined the correlation between the expression of the four hub genes and immune cell infiltration levels in BRCA. As depicted in Figure 4A, the expression of the four hub genes was closely associated with the most TIICs abundance in BRCA patients, which also suggested that the four hub genes were closely associated with immunity. The survival analysis of the four hub genes was further analyzed (Figures 4B–E). The results show that the overexpression of SPI1 and SELL due to pDC abundance was significantly associated with good clinical outcomes in BRCA patients (*p* < 0.05). Another two hub genes (CD34 and IL6) showed no significant prognostic value.

In addition, the pan-cancer expression of the hub genes was further analyzed, and the data on the hub gene expression were extracted from the TIMER database, which is an in-depth resource for systematic analyses across multiple malignancies. Among the results, the expression levels of CD34, IL6, SPI1, and SELL showed significant expression differences in 16, 12, 12, and 11 cancer types, respectively, especially in BRCA (Supplementary Figure 4).

### Analysis of Copy Number Aberrations Between the pDC Groups

**FIGURE 5:**
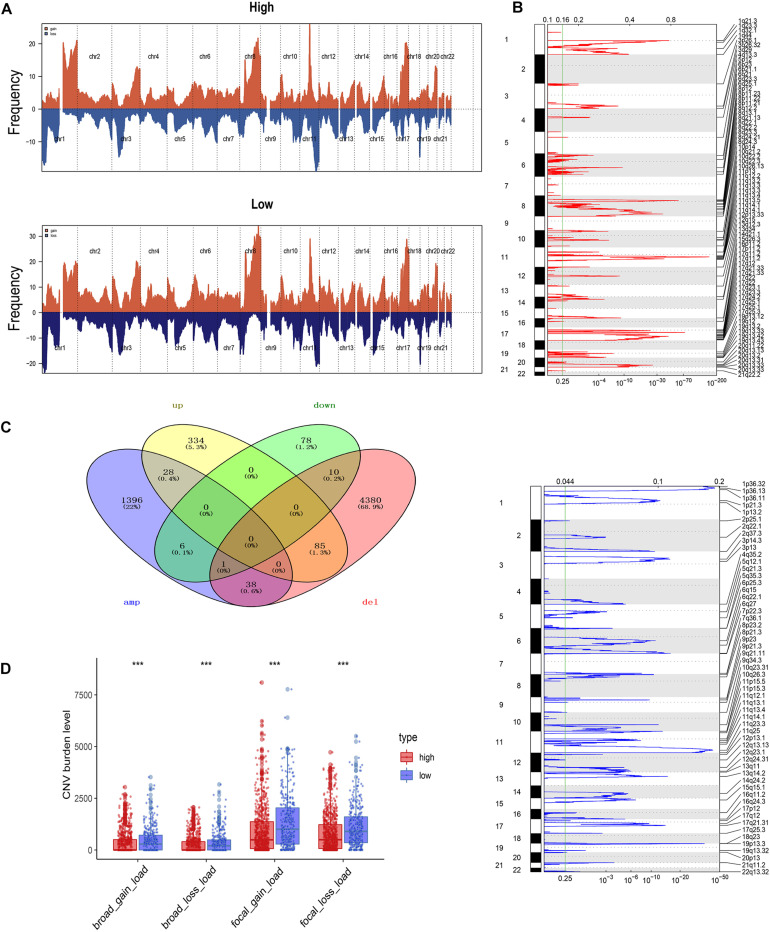
Somatic copy number aberration across the pDC groups. **(A)** The copy number amplifications and deletions between the two groups. **(B)** Detailed cytoband with focal amplification (top) and focal deletion (bottom) in the pDC^high^ group generated with GISTIC_2.0. **(C)** Venn diagram of the genes with SCNA and the DEGs in the pDC^high^ group. **(D)** The burden of SCNA between the pDC^high^ group and the pDC^low^ group. The meaning of the statistical difference is as follows: *** represents *p* ≤ 0.001.

Using the GISTIC 2.0 module of GenePattern, we also assessed the difference in alterations in somatic copy numbers between the pDC^high^ group and the pDC^low^ group. The 22 chromosomes in the two groups exhibited a similarity in chromosomal aberrations, and the distribution of the frequency across all chromosomes in these groups is shown in Figure 5A. As is shown in Figure 5B, many focal amplifications (e.g., 6p21.1, 8q23.3, and 11q12.2) and deletions (e.g., 1p36.13, 11q23.3, and 19p13.3) within chromosomal regions were detected in the higher pDC abundance group. In addition, we compared the DEGs with copy number aberrations in the high pDC abundance group, and only a few genes showed a similar mode with its expression level as shown in Figure 5C. This implies that most of the DEGs were not affected by SCNA. In other words, differential gene expression between the pDC^high^ group and the pDC^low^ group occurred without the effects of SCNA.

To evaluate the impact of SCNA on the pDC, we examined the differences of the burden of copy number alterations in arm level and focal level between these distinct groups (Figure 5D). Compared with those in the pDC^high^ group, the pDC^low^ group demonstrated a higher burden of SCNA in focal and arm levels. These results above suggested that SCNA contributed to differences in immune cell infiltration in BRCA, and the patients of the pDC^high^ group may be more sensitive to immunotherapy.

### Differential Somatic Mutation Landscape Between the pDC Subgroups

**FIGURE 6:**
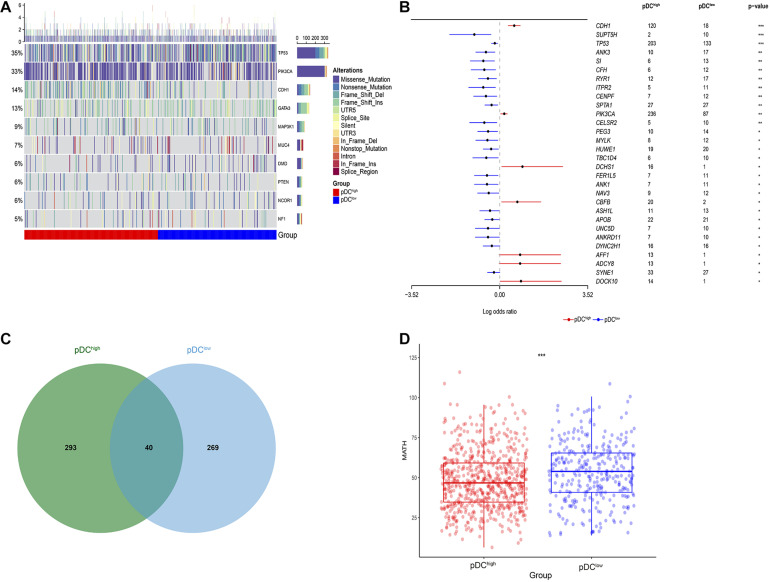
Genetic alteration across the pDC groups. **(A)** Somatic mutation landscape of significantly mutated genes in TCGA. **(B)** Differentially mutated genes between the pDC^high^ group and the pDC^low^ group. **(C)** Shared significantly mutated genes between the pDC^high^ group and the pDC^low^ group. **(D)** Boxplot of intratumoral heterogeneity between the two groups. The meaning of the statistical difference is as follows: * represents *p* ≤ 0.05, ** represents *p* ≤ 0.01, and *** represents *p* ≤ 0.001.

After analyzing the transcriptional alterations and copy number aberrations between the two groups, we further investigated the differences in somatic mutations between the pDC^high^ group and the pDC^*low*^ group. Somatic mutations data were downloaded from TCGA portal using the R package TCGAbiolinks. By MutSigCV_v1.41 tools, among all BRCA samples, we identified 10 significantly mutated genes, including TP53, PIK3CA, CDH1, GATA3, MAP3K1, MUC4, DMD, PTEN, NCOR1, and NF1, after removing genes whose non-silent mutation rate was less than 5% (Figure 6A). Additionally, 30 differentially mutated genes between the two groups were identified by using the mafCompare() function in the R package “maftools” (Figure 6B). Additionally, 3 of these differentially mutated genes, including TP53, PIK3CA, and CDH1, were consistent with 10 significantly mutated genes among all BRCA samples above. In addition, we determined 333 and 309 significant mutation genes by MutSigCV for the pDC^high^ group and the pDC^low^ group, respectively, at a loose cut-off of *p* < 0.05, and only 40 significant mutation genes were shared (Figure 6C).

Moreover, the intratumoral heterogeneity of each sample was determined by the MATH value and was the ratio of MAD to the median of mutant-allele fractions. For MATH data, the difference between the pDC^high^ group and the pDC^low^ group was determined using the Wilcoxon test (Mann–Whitney test). There was a significant difference between the two groups (*p* < 0.001), and lower intratumoral heterogeneity existed in the pDC^high^ group, as shown in Figure 6D.

### Differential Sensitivity Analysis of Immuno/Chemotherapies Between the pDC Groups

**FIGURE 7:**
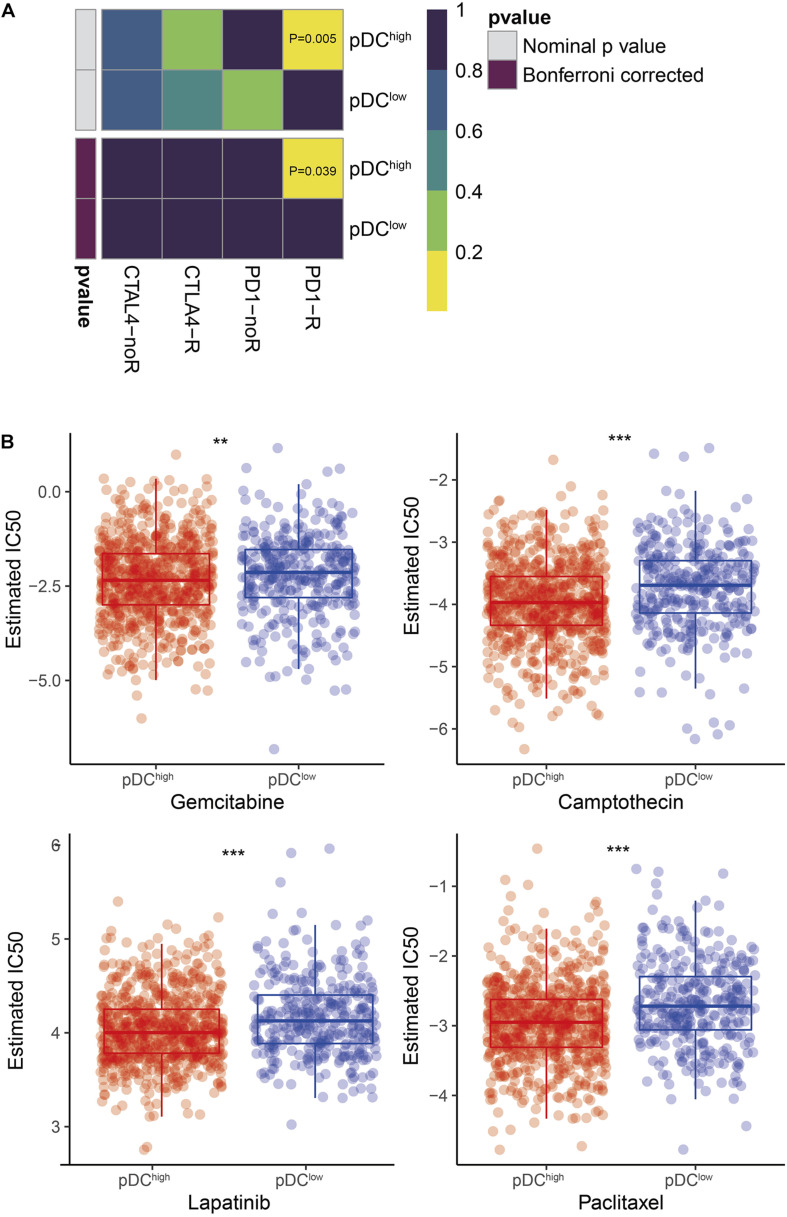
Differential putative immunotherapeutic and chemotherapeutic response prediction. **(A)** Submap analysis in two groups. **(B)** The box plots of chemotherapeutic response in two groups. The meaning of the statistical difference is as follows: ** represents *p* ≤ 0.01 and *** represents *p* ≤ 0.001.

According to our knowledge, the effect of immunotherapy is closely related to the burden of SCNA and intratumoral heterogeneity. Then, the likelihood of immunotherapeutic responses was determined using two methods. First, using the subclass mapping method, we compared the expression profiles of the two groups with a dataset of 47 melanoma patients who responded to immunotherapy. It demonstrated that the higher pDC group was more sensitive to anti-PD-1 therapy (*p* = 0.039) (Figure 7A). The likelihood of immunotherapeutic responses was also predicted using the TIDE algorithm. Significant differences between the two groups were shown (*p* < 0.01). According to previous results, it also was consistent that patients with a lower burden of somatic copy number variations (SCNV) and lower intratumoral heterogeneity in the pDC^high^ group were more sensitive to immunotherapy.

In BRCA clinical practice, chemotherapy is a common method to treat the tumor. Thus, four chemo drugs, gemcitabine, camptothecin, lapatinib, and paclitaxel, were used to assess the different responses to chemotherapy between the two pDC groups. Firstly, cell line data from the GDSC database were downloaded. Then, using the ridge regression, we trained a predictive model method and determined the predictive accuracy by 10-fold cross-validation. We calculated the IC50 values for gemcitabine, camptothecin, lapatinib, and paclitaxel of each patient by the predictive model. Finally, we found that there was a significant difference in sensitivity to gemcitabine, camptothecin, lapatinib, and paclitaxel, as shown in Figure 7B.

### Demographic Characteristics

According to the cut-off point confirmed previously, 751 cases were classified into the pDC^high^ group and 346 into the pDC^low^ group. Then, we performed a series of chi-square tests to inspect the correlation between the abundance of the pDC and clinicopathological characteristics. Most of the clinicopathological characteristics, including patient age, gender, and pathological stage, were not different between the two groups, except M stage, N stage, and T stage (Supplementary Table 1). Concurrently, univariate and multivariate Cox regression analyses were conducted to assess whether the prognostic ability of the pDC was independent of other clinical features. The result of univariate Cox regression indicated that the risk score was significantly associated with OS [pDC^low^ group vs. pDC^high^ group, hazard ratio (HR) = 1.579, 95% CI 1.143–2.182, *p* < 0.01]. Additionally, in multivariable Cox regression, the pDC have a significant relationship with OS (pDC^low^ group vs. pDC^high^ group, HR = 1.570, 95% CI 1.131–2.178, *p* < 0.01) (Supplementary Table 2). These results demonstrated that the prognostic ability of the pDC was independent of other clinical features. In addition, the relationship between clinical features (tumor stage and age) and pDC abundance was explored. The analyzed results suggested that pDC abundance showed no significant difference in tumor stage and age, which is consistent with previous results in Supplementary Table 1 (Supplementary Figure 5).

## Discussion

DCs are the most important antigen-presenting cells (APC). The migration ability of immature DCs has been documented to be strong. Mature DCs effectively activate initial T cells and regulate and maintain the central link of the immune response. This maturation occurs in tandem with antigen uptake, processing, as well as presentation to activate T cells. Otherwise, DCs induce antigen-specific T cell tolerance or silencing. On the one hand, the secondary products of the pDC, especially type I IFNs, exhibit both tolerogenic and immunogenic effects in tumor immunity. These factors enhance the cytotoxicity of T cells and NK cells, help activate B cells to differentiate into plasma cells, enhance the maturation and activation of DCs as well as pro-inflammatory macrophages, and finally jointly contribute to an immune-activated TME. On the one hand, the pDC also recruit Treg cells or induce the expression of immune regulatory molecules to create an immunosuppressive TME.

The complex roles of the pDC allow them to perform different functions in BRCA. It has been documented that, through TRAIL and Granzyme B, the activated pDC can kill breast tumor cells. Furthermore, the pDC initiate the sequential activation of CD8 + T cells as well as NK cells and ultimately suppress BRCA growth (Wu et al., 2017). Kini studied 75 newly diagnosed BRCA patients aged 28–87 and found a positive correlation between higher than median levels of circulating pDC and 5-year survival (Kini Bailur et al., 2016). While another study has revealed that pDC infiltration would lead to poor prognosis because the pDC promote lymph node metastasis *via* the CXCR4/SDF-1 axis (Gadalla et al., 2019). These studies prove that the pDC can predict the prognosis of BRCA, but the specific role is still controversial. Therefore, we determined the potential role of the pDC-associated genes in BRCA. The abundance of the pDC was calculated by the ssGSEA algorithm in three cohorts. In this research, BRCA samples were assigned into two groups based on the abundance of the pDC. An elevated abundance of the pDC was correlated with better survival outcomes in BRCA patients. Concurrently, this conclusion also was confirmed by another two external cohorts.

Then, further analysis identified 542 DEGs between the groups. KEGG pathway annotation and GO annotation were conducted by the GSEA algorithm, and the results indicated that the pDC^high^ group was more related to immune response. In order to select the hub genes associated with the pDC, the PPI network was conducted through the STRING database, three subnetworks were filtered out, and finally, we identified four hub genes. Among the four hub genes, we also found that two hub genes (SELL, SPI1) related to the pDC have independent prognostic significance. These two genes are potential prognostic biomarkers with therapeutic implications. Selectin is a type of Ca^2+^-dependent cell adhesion molecule, which can recognize and bind to specific glycosyl groups, involved in the recognition and adhesion between white blood cells and vascular endothelial cells. The selectin family has three members: E-selectin, L-selectin, and P-selectin. L-selectin (SELL) was first discovered as a homing receptor on lymphocytes and later found to be expressed on various white blood cells. Chu et al. documented that SELL immunodepletion inhibited MDA-MB-231 cell migration (Laubli and Borsig, 2010; Geng et al., 2013; Chu et al., 2014). SPI1 transcription factors are closely related to developmental processes. It is a major regulator hematopoiesis and limits hematopoietic stem cell self-renewal. Deregulation of its activity or expression is associated with leukemia, in which SpI1 can act as either an oncogene or a tumor suppressor. Delestre et al. (2017) documented that senescence is an anti-proliferative mechanism induced by SpI1 that may inhibit the pathogenesis of acute myeloid leukemia. Moreover, the stemness of T-ALL leukemia stem cell is epigenetically regulated by SPI1 (Zhu et al., 2018). In addition, the expression of SpI1 can promote the early myeloid development of zebrafish (Yu et al., 2018). Although the other two genes (IL6, CD34) did not show significant prognostic value, they are still crucial for the occurrence and development of BRCA. Interleukin 6 (IL6)/STAT3 signaling enhances BRCA metastasis by interfering with estrogen receptor (ER) alpha (Hu et al., 2020; Siersbaek et al., 2020). The expression of CD34 in BRCA tissues is positively correlated with angiogenesis, and its high expression will promote cancer cell infiltration and metastasis (Shpall et al., 1994; Schulman et al., 1999; Westhoff et al., 2020).

SCNA analysis also was performed by the GISTIC 2.0 module of GenePattern. There was clear similarity between chromosomal aberrations in two groups. However, the burden of copy number alterations in arm level and focal level also was calculated, and the pDC^high^ group exhibited a lower burden of SCNV that could contribute to the difference in immunotherapy response between the two groups. Furthermore, the genomic analysis revealed a distinct mutation landscape. In this paper, 3 differentially mutated genes, including TP53, PIK3CA, and CDH1, were overlapped with 10 significantly mutated genes among all BRCA samples. The differentially mutated gene TP53 was discoverd in the pDC^high^ group. TP53 is the tumor suppressor gene with the highest correlation with human tumors discovered so far. More than 50% of tumor patients have p53 gene mutations. In the cell cycle, normal p53 is activated when DNA damage or hypoxia causes the cell cycle to stagnate in the G1/S phase and perform DNA repair. Failure to repair will activate downstream genes to start cell apoptosis. PIK3CA gene is responsible for encoding the p110α protein, which is a subunit of the PI3K enzyme. The main function of the PI3K enzyme is phosphorylation, which triggers a series of intracellular signal transmission through phosphorylation of other proteins. These signals are related to cell activities, including cell proliferation, migration, survival, as well as new protein production and intracellular material transport. The pathway that PI3K participates in is the PI3K–AKT–mTOR pathway. Pathogenic mutations in PIK3CA will cause it to encode abnormal p110α subunits, which will keep the PI3K enzyme in a state of continuous activation, which enhances the signal transduction in the cell, leading to uncontrolled cell proliferation and thus the formation of tumors. The classic cadherin of the cadherin superfamily is encoded by the *CDH1* gene. Alternative splicing can lead to multiple transcriptional variants. This calcium-dependent intercellular adhesion protein is composed of a transmembrane region, five extracellular cadherin repeats, and a cytoplasmic tail that is highly conserved. It has been documented that mutations affecting the *CDH1* gene result in several types of cancers including ovarian, thyroid, colorectal, breast, and gastric cancers. Loss of function of *CDH1* promotes the progression of cancers and metastasis.

In conclusion, to the best of our knowledge, this is the first comprehensive differential molecular profiling of BRCA that is based on the abundance of the pDC. Molecular differences may be beneficial in aiding in the development of immunotherapy and opened up a new world for the precision treatment of BRCA. However, this study is associated with some limitations. First, the conclusion is based on *in silico* analysis only. Prospective population-based studies should be performed to verify our findings. Second, a larger sample size should be used to identify the potential differences in clinicopathological features. Finally, the response to chemotherapy is just based on the GDSC dataset, and more datasets should be used to validate the observation in future work.

## Data Availability Statement

Publicly available datasets were analyzed in this study. This data can be found here: https://portal.gdc.cancer.gov, https://www.ncbi.nlm.nih.gov/geo.

## Author Contributions

ST, LY, and LF contributed equally to this article, collected and analyzed data, and drafted and revised the manuscript. WZ designed the study. ZZ, JZ, and GM collected data and revised the manuscript. All authors read and approved the final manuscript.

## Conflict of Interest

The authors declare that the research was conducted in the absence of any commercial or financial relationships that could be construed as a potential conflict of interest.

## Footnotes

1 https://portal.gdc.cancer.gov

2 http://www.firehose.org/

3 https://www.ncbi.nlm.nih.gov/geo

4 http://string-db.org

5 http://www.firehose.org/

6 www.broadinstitute.org

7 https://www.cancerrxgene.org/

8 http://www.cytoscape.org/

## Supplementary Material

Supplementary Figure 1 The PPI network for all DEGs.

Supplementary Figure 2 The three subnetworks of PPI according to **(A)** degree, **(B)** betweenness, and **(C)** closeness.

Supplementary Figure 3 Venn diagram of three subnetworks.

Supplementary Figure 4 CD34, IL6, SPI1, and SELL expression in different cancer types. **(A–D)** The expression levels of CD34, IL6, SPI1, and SELL in TCGA are shown. The meaning of the statistical difference is as follows: ns represents not significant (*p* > 0.05), ^∗^ represents *p* ≤ 0.05, ^∗∗^ represents *p* ≤ 0.01, and ^∗∗∗^ represents *p* ≤ 0.001.

Supplementary Figure 5 The relationship between clinical features and pDC abudance. **(A)** Tumor stage. **(B)** Age.

Supplementary Table 1 Demographic characteristics of two groups.

Supplementary Table 2 Univariate and multivariate Cox regression analyses of the pDC in TCGA set. Abbreviations: HR, hazard ratio; CI, confidence interval.
