# Supplementary figures and images for: A Comprehensive Investigation to Reveal the Relationship Between Plasmacytoid Dendritic Cells and Breast Cancer by Multiomics Data Analysis
Source: Front Cell Dev Biol. 2021 Apr 1;9:640476. doi: 10.3389/fcell.2021.640476 (PMC8047150; doi:10.3389/fcell.2021.640476)

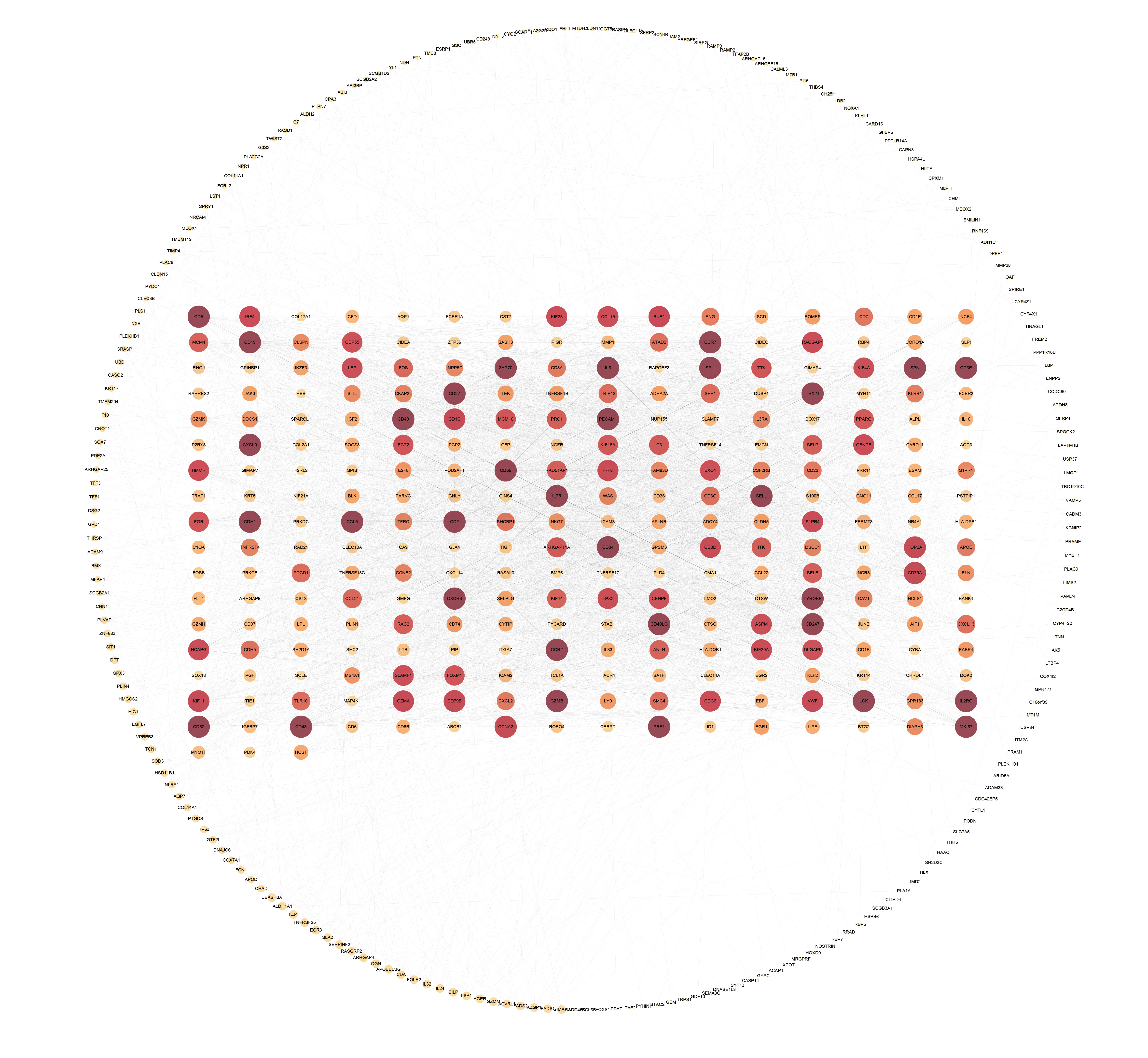

Supplement: Supplementary Figure 1 — The PPI network for all DEGs. [file Image_1.JPEG]

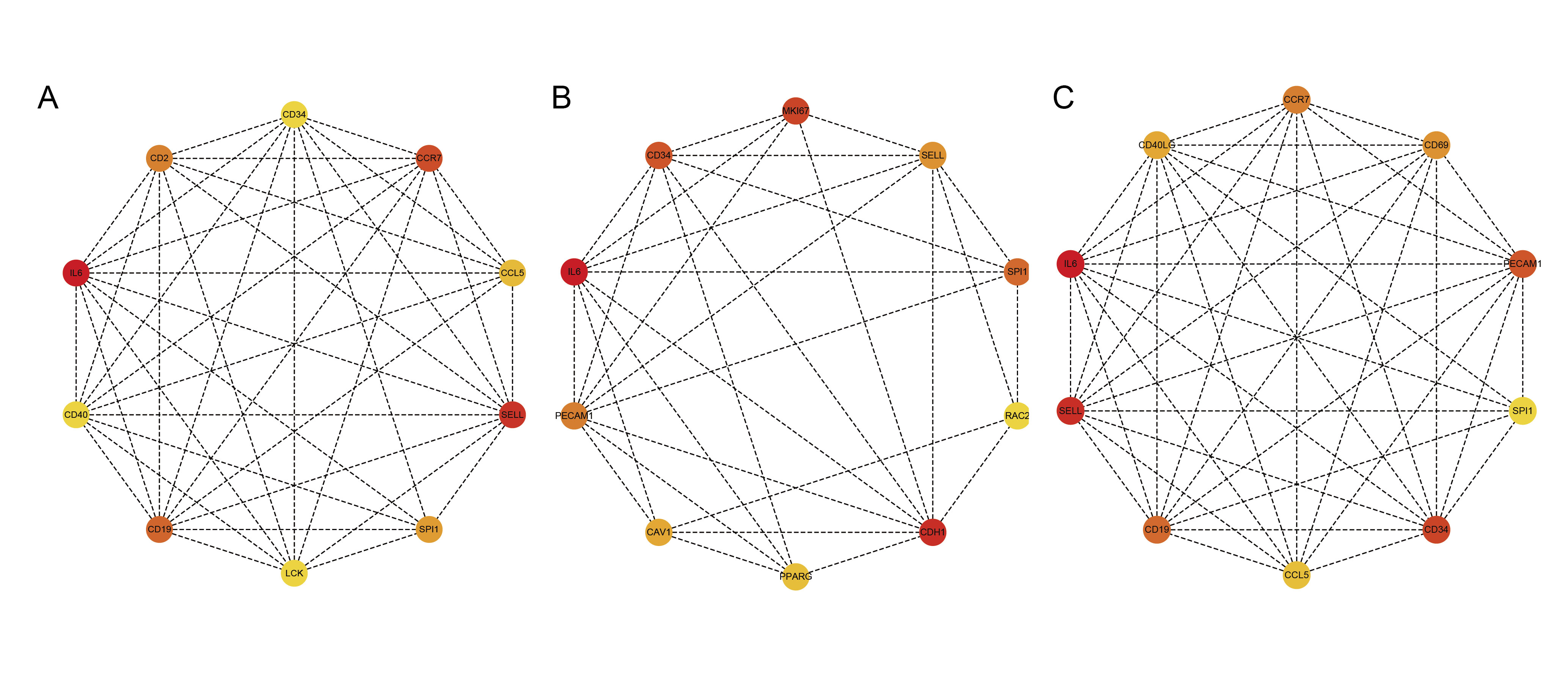

Supplement: Supplementary Figure 2 — The three subnetworks of PPI according to (A) degree, (B) betweenness, and (C) closeness. [file Image_2.JPEG]

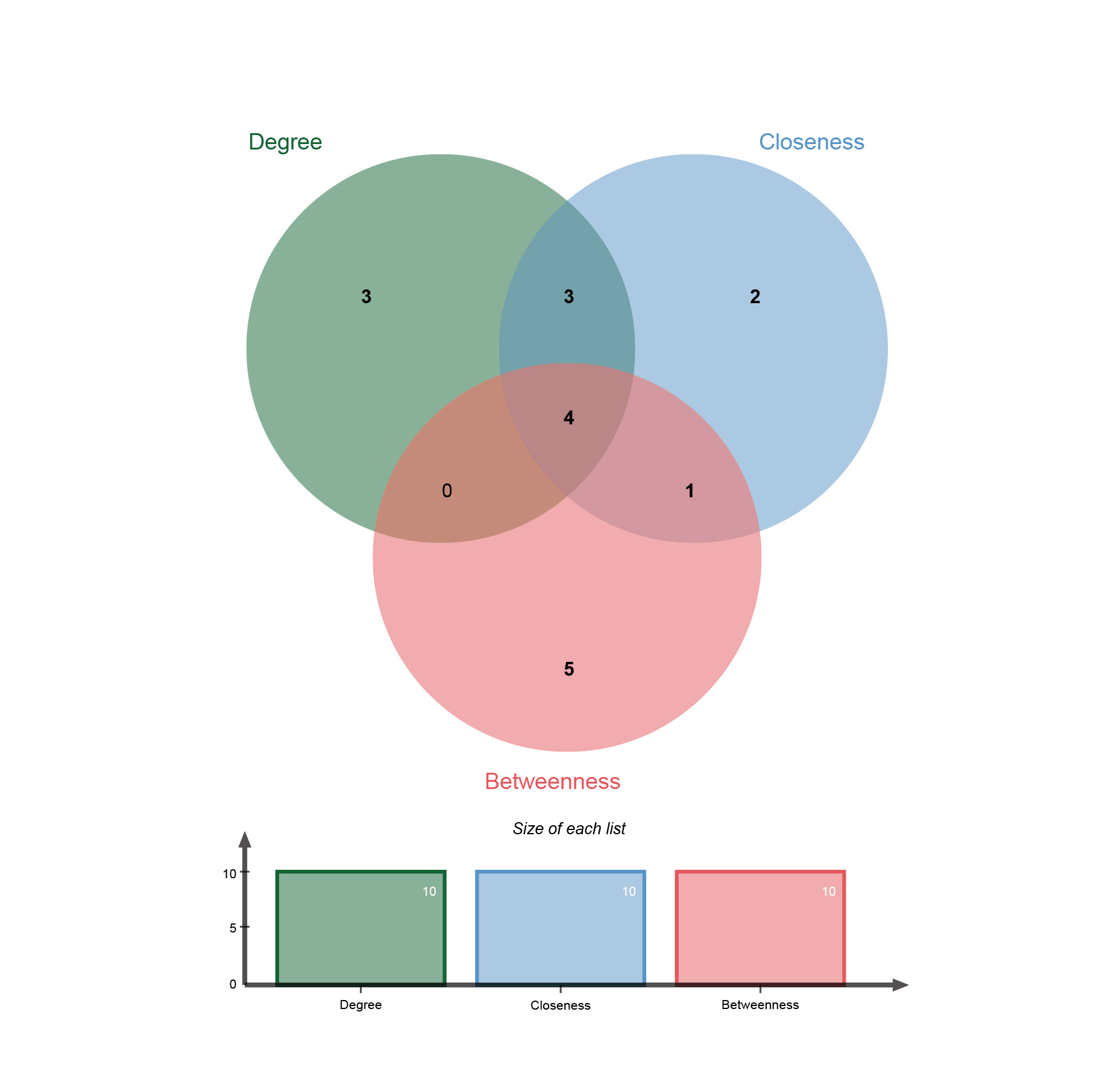

Supplement: Supplementary Figure 3 — Venn diagram of three subnetworks. [file Image_3.JPEG]

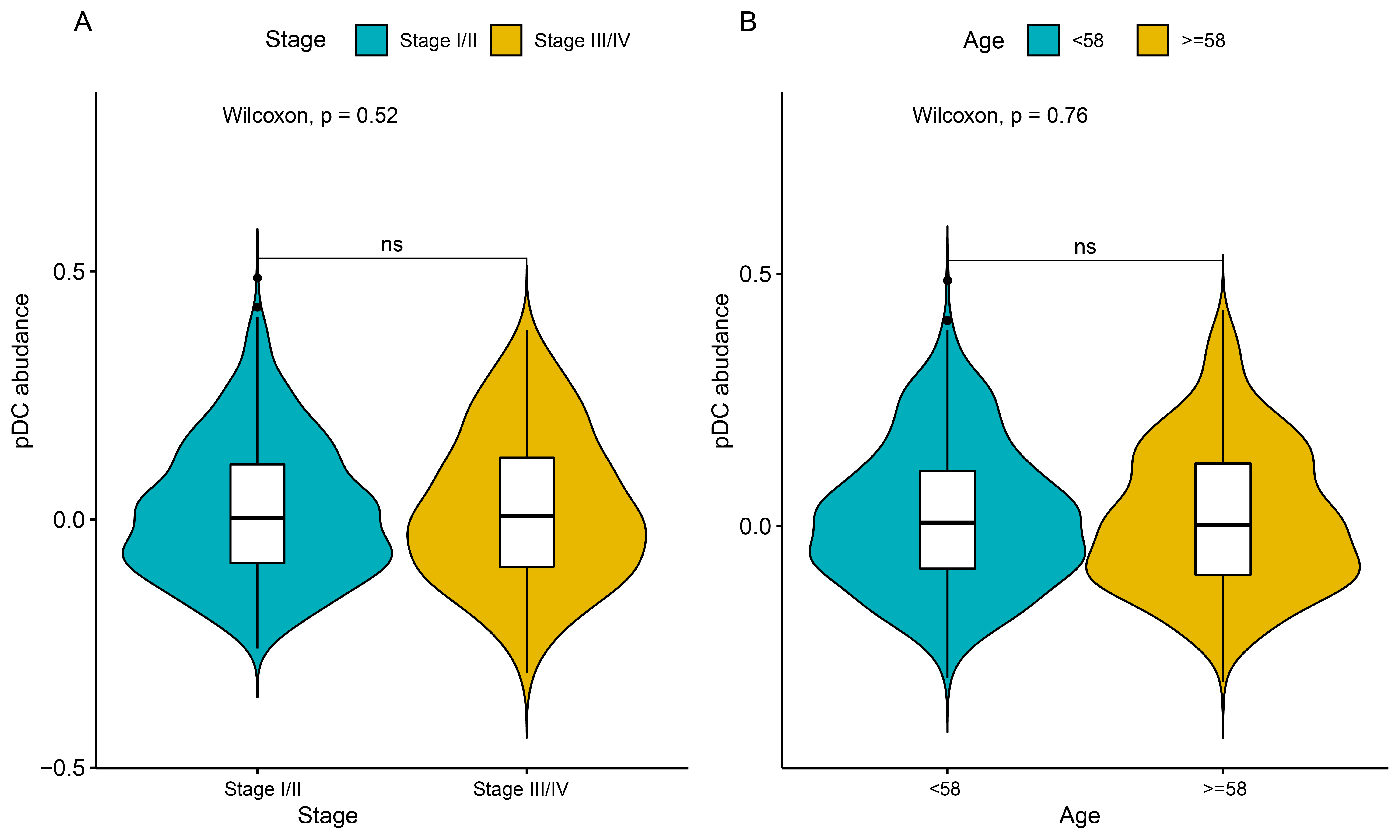

Supplement: Supplementary Figure 5 — The relationship between clinical features and pDC abudance. (A) Tumor stage. (B) Age. [file Image_5.JPEG]
