# Supplementary material for: A Comprehensive Investigation to Reveal the Relationship Between Plasmacytoid Dendritic Cells and Breast Cancer by Multiomics Data Analysis
Source: Front Cell Dev Biol. 2021 Apr 1;9:640476. doi: 10.3389/fcell.2021.640476 (PMC8047150; doi:10.3389/fcell.2021.640476)
Supplement: Supplementary Table 1 — Demographic characteristics of two groups. [file Data_Sheet_1.PDF]

Table S1. Demographic characteristics between two groups.

Variables	pDC		P
	pDC^{high}	pDC^{low}	
**Gender**			0.117
Female	740	345	
Male	11	1	
**Age**			0.144
>=58	360	183	
<58	391	163	
**M stage**			0.001
M0	601	306	
M1	150	40	
**N stage**			0.003
N0	340	174	
N1	266	99	
N2	77	43	
N3	60	18	
NX	8	12	
**T stage**			0.004
T1	202	78	
T2	419	218	

T3	107	30	
T4	22	18	
**pTNM stage**			0.144
Stage I	127	54	
Stage II	423	202	
Stage III	179	71	
Stage IV	13	7	
Stage X	5	8	

Table S2 Univariable and multivariable cox regression analysis of the pDC in the TCGA set. Abbreviations: HR, hazard ratio; Cl, confidence interval.

**The TCGA set**					
**Variables**		**HR**	**95% CI of HR**		**P**
			**lower**	**upper**	
**Univariate analysis**					
Age	>=58 vs. <58	1.712	1.244	2.356	0.001
Gender	Male vs. Female	0.832	0.116	5.956	0.854
pTNM stage	III/IV vs. I/II	2.602	1.893	3.576	0.000
M stage	M1vs. M0	1.868	1.256	2.778	0.002
T stage	T3/4 vs. T1/2	1.596	1.113	2.291	0.011
N stage	N2/3 vs. N0/1	2.511	1.78	3.541	0.000
pDC	pDC^{low} vs. pDC^{high}	1.579	1.143	2.182	0.005
**Multivariable analysis**					
Age	>=58 vs. <58	1.724	1.249	2.379	0.001
Gender	Male vs. Female	0.853	0.118	6.155	0.875
pTNM stage	III/IV vs. I/II	2.281	1.298	4.011	0.004
M stage	M1vs. M0	1.364	0.899	2.068	0.144
T stage	T3/4 vs. T1/2	0.836	0.525	1.331	0.450
N stage	N2/3 vs. N0/1	1.332	0.791	2.245	0.282
pDC	pDC^{low} vs. pDC^{high}	1.570	1.131	2.178	0.006
